# Managing Conservation Reliant Species: Hawai'i's Endangered Endemic Waterbirds

**DOI:** 10.1371/journal.pone.0067872

**Published:** 2013-06-25

**Authors:** Jared G. Underwood, Mike Silbernagle, Mike Nishimoto, Kim Uyehara

**Affiliations:** 1 Pacific Reefs National Wildlife Refuge Complex, U.S. Fish and Wildlife Service, Honolulu, Hawai'i, United States of America; 2 Oahu National Wildlife Refuge Complex, U.S. Fish and Wildlife Service, Haleiwa, Hawai'i, United States of America; 3 Maui National Wildlife Refuge Complex, U.S. Fish and Wildlife Service, Kihei, Hawai'i, United States of America; 4 Kauai National Wildlife Refuge Complex, U.S. Fish and Wildlife Service, Kilauea, Hawai'i, United States of America; University of Kent, United Kingdom

## Abstract

Hawai'I's coastal plain wetlands are inhabited by five endangered endemic waterbird species. These include the Hawaiian Coot ('alae ke'oke'o), Hawaiian Duck (koloa maoli), Hawaiian Stilt (ae'o), Hawaiian Gallinule (Moorhen) ('alae 'ula), and Hawaiian Goose (nēnē). All five species are categorized as being “conservation reliant.” The current strategy to recover these endangered birds includes land protection and active management of wetlands. To assess the effectiveness of the current management paradigm, we compared species population trends across the state to those on six actively managed wetland national wildlife refuges (Refuges) thought to be critical for the survival of these endangered species. To perform the evaluation we relied on systematic semiannual population counts that have been conducted across most wetlands in the state and monthly population counts that have occurred on Refuges during the same time period. We found that statewide and Refuge populations of the Hawaiian Coot, Stilt and Gallinule have rebounded from historic lows and over the last 20 years have slowly increased or remained stable. We also documented that Refuges are important to each species year-round and that a disproportionately larger percentage of the population for each species is found on them. Understanding of why Refuges successfully house a disproportionate percentage of these “conservation reliant” species can inform current and future conservation efforts as well as ensure long-term population viability for these species.

## Introduction

Hawai'i is home to more endangered species than any other state in the United States [1]. A quarter of all species listed under the United States Endangered Species Act of 1973, including one-third of all birds listed, are native to Hawai'i [1]. Listed species in Hawai'i include five endangered endemic waterbirds, the Hawaiian Coot ('alae ke'oke'o; *Fulica alai*), Hawaiian Duck (koloa maoli; *Anas wyvilliana*), Hawaiian Stilt (ae'o; *Himantopus mexicanus knudseni*), Hawaiian Gallinule (Moorhen) ('alae 'ula; *Gallinula galeata sandvicensis*), and Hawaiian Goose (nēnē; *Branta sandvicensis*). Endangerment of these five species has been attributed to a loss or modification of wetland habitats, introduced plants and predators, avian diseases, altered hydrology, hybridization, and historic overhunting [2], [3]. Faced by this suite of cumulative threats, these endangered Hawaiian birds have recently been categorized as “conservation reliant”, which implies that they will require active management into perpetuity [4], [5], [6].

These endangered waterbirds use a variety of wetland and open water environments in Hawai'i including freshwater/saltwater marshes and ponds, estuaries and mudflats, artificial reservoirs, agricultural lands (primarily taro-*Colocasia esculenta*- and stock watering ponds), irrigation and flood control ditches, sewage treatment ponds, and in the case of the Hawaiian Duck, montane streams and bogs [2]. How each species utilizes this system of wetlands and the interconnectedness of the wetland landscape is poorly understood. Today, approximately 6,190 ha of lowland coastal plain wetlands, the wetland type most commonly used by the endangered waterbirds, persist in the state [2], [7]. This represents a 31% reduction from the total estimated in 1780, with the majority of remaining coastal plain wetland areas degraded by altered hydrology, invasive species, and contaminants [2], [7]. It has generally been proposed that birds disperse across these wetlands during the rainy season (October- March) and congregate in key wetlands during the dry season (April-September) [2], [3].

For the past several decades, the accepted active management strategy for these species has included three major components [2], [8]. First is the control of invasive introduced plant species. Hawai'i experiences a year-round growing season and therefore management of invasive wetland plants must be constant. When wetlands are not actively managed, waterbird habitat can rapidly degrade as weedy invaders tend to fill open water and mudflat areas important to the birds. When combined with altered hydrology and sedimentation, wetlands can rapidly become completely unsuitable for endangered waterbirds. Control methods for invasive plants include herbicides, mechanical treatment, manipulation of water levels, and prescribed fire. Second is the control of introduced predators. Non-native cats, rats, mongooses, dogs, and to a lesser extent wild pigs, Barn Owls (*Tyto alba*), Cattle Egrets (*Bubulcus ibis)*, predatory fish, and bullfrogs all directly depredate either eggs, young, or adult birds. To combat the mammalian predators that have the greatest negative impact, wetlands must have a barrier of fences, rodenticide baited stations, and live or kill traps around the areas used by waterbirds. Depredation rates and population-level effects of the non-mammalian predators mentioned, as well as effective control methods, are still being investigated. Third is the manipulation of wetland water levels to mimic natural hydrological processes that have been highly altered or lost due to anthropogenic impacts. Water control in wetlands is facilitated by a system of dikes and levees with water pumped from wells or procured from permanent free-flowing water sources (i.e., rivers, streams). This allows wetlands to maintain water levels in times of drought and to adjust water levels to best suit the needs of breeding birds. Although all three of these management actions may not occur at the same time/location, it is generally accepted by wetland managers in Hawai'i that all three management actions in concert are required to begin to restore the functionality of wetlands to meet the life-history requirements of these birds [2], [8]. While the core components of the current active management strategy have remained the same, methods and tools have changed and will continue to change as managers face new threats and refine techniques for Hawai'i's wetland systems.

Recovery (downlisting or delisting from the endangered species list) for the Hawaiian Stilt, Coot, Gallinule and Duck is linked to the permanent protection and active management of key remaining coastal plain wetlands [2]. Six coastal plain wetland national wildlife refuges (Refuges) on the main Hawaiian Islands have all been identified as core wetlands that will contribute toward recovery [2], [9]. These Refuges have some of the most actively managed of Hawai'i's wetlands. In this study we use Refuges to evaluate the effectiveness of the current management paradigm for these conservation reliant species.

To assess the importance of Refuges and their associated management practices to waterbird populations, we evaluated three metrics: long-term population trends, percentage of the population for each species found on Refuges over time, and seasonal use of Refuges. To assess long-term population trends, we analyzed and compared statewide population trends (all wetlands), Refuge specific trends, and non-Refuge wetland trends. In our analysis, non-Refuge wetlands refer to all surveyed wetlands other than the aforementioned six Refuges. These non-Refuge wetlands include some wetlands that are currently actively managed for waterbirds and others that have no active management. The analyses we performed, lumped managed and unmanaged non-Refuge wetlands. If the active management program on Refuge wetlands is an effective strategy, we might expect to document an enhanced population trend on Refuges compared to non-Refuge wetlands. Since we did not include all actively managed wetlands in our Refuge category, this expectation may not hold true as active management on non-Refuge wetlands may also have benefited these bird species. Second, we calculated the percentage of the population for each species that is found on the Refuges. This allowed for an evaluation of the relative importance of Refuge wetlands using bird population densities. If the active management program on Refuge wetlands is an effective strategy, we would expect to find proportionally higher bird populations on Refuges compared to wetland areas off Refuge. Lastly, we assessed the seasonal use of Refuges by each species to determine if these wetlands were important year-round for the birds. If the active management program on Refuge wetlands is an effective strategy, we would expect year-round use of Refuges rather than the seasonal use observed in non-Refuge wetlands.

## Methods

### Study Area

**Figure 1:**
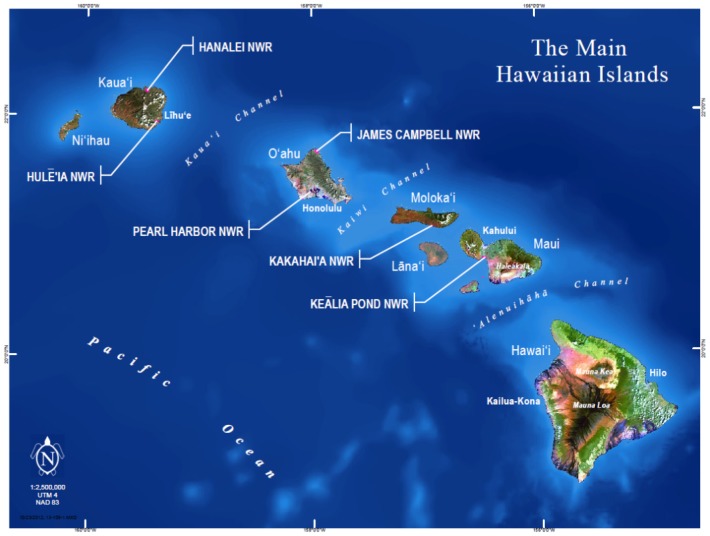
Map of “Main Hawaiian Islands” with the six coastal plain national wildlife refuges identified. Hawai'i, USA.

The endangered waterbird species are currently found on various “main Hawaiian Islands” (18°54′N to 22°12′N, 154°54′W to 160°12′W; Fig. 1). The term “main Hawaiian Islands” refers to the following eight islands: Ni'ihau, Kaua'i, O'ahu, Maui, Moloka'i, Lāna'i, Kaho'olawe, and Hawai'i. Semiannual waterbird surveys currently occur on all main Hawaiian Islands except for Ni'ihau and Kaho'olawe. The six coastal plain wetland Refuges occur across the main Hawaiian Islands (see Figure 1).

### Coastal Plain Wetland Extent

The extent of coastal plain wetlands in the main Hawaiian Islands is approximately 6,190 ha. It should be noted that this estimate of wetlands does not include all areas used by the birds as not all wetlands such as reservoirs, irrigation and flood control ditches, golf course water features, streams, ephemeral springs and basins, or sewer treatment ponds are included. Of the coastal plain wetlands, 3,500 ha are identified as important for the recovery of waterbirds [2]. The six Refuges contain approximately 900 ha or 15% of all coastal plain wetlands and 26% of those identified as important to waterbirds. The 900 ha of wetlands on Refuges are as follows: Hanalei National Wildlife Refuge (NWR) (371 ha), Kealia Pond NWR (280 ha), James Campbell NWR (107 ha), Huleia NWR (98 ha), Pearl Harbor NWR (25 ha), and Kakahai'a NWR (18 ha) [2].

### Species

For our analysis we focused on the Hawaiian Coot, Gallinule, and Stilt. We did not include the Hawaiian Duck in our analysis because population count results are complicated by the presence of feral Mallard (*Anas platyrhynchos*) and Hawaiian Duck hybrids. It can be difficult if not impossible to distinguish a pure Hawaiian Duck from a Mallard-Hawaiian Duck hybrid in the field, especially females. In addition, streams are not included in the surveys, which is where the majority of Hawaiian Ducks are believed to reside [2]. These factors make use of the state semiannual population counts for this species unreliable. We also did not incorporate the Hawaiian Goose as their primary habitats are in dry uplands and not coastal wetlands, although wetlands and other water bodies may be used during their annual cycle [10].

### Data- Semiannual State Counts & Monthly Refuge Counts

Semiannual waterbird population surveys have been conducted statewide since the mid-1950s, but coverage was somewhat inconsistent until the 1980s. Currently, each census consists of simultaneous visits to wetlands on all islands during a single day or two-day time period in January and August [3], [11]. This is done to reduce the possibility of double counting birds. These surveys include the majority of wetlands on each island, but do not cover all locations that support waterbirds such as streams, flood control ditches, and some private lands. During a survey, all waterbirds observed or heard on each wetland are counted. The counts resulting from these surveys are considered a minimum population estimate and certainly underestimate the actual population. Population estimates resulting from these surveys have not been corrected for detectability or observer differences/abilities. For the Hawaiian Coot and Stilt this probably does not have a significant impact as both species often use open water and mudflat areas and are considered to have detectability approaching 100% [2]. The Hawaiian Gallinule however, is secretive and often hides in densely vegetated areas resulting in detectability problems that lead to undercounting [2], [12], [13]. Even with these limitations, since the methodology has remained consistent over time, we can still detect changes in relative abundance. During each census approximately 400 locations are surveyed, which include the remaining natural coastal plain wetlands as well as some anthropogenic wetlands (i.e., golf course ponds, waste treatment ponds, reservoirs). Monthly or bimonthly waterbird censuses have also been conducted on the six Refuges for varying lengths of time. To evaluate seasonal use patterns, we used data from five of the six Refuges. Data collected on Kakahai'a NWR only existed outside the timeframe analyzed. The methods employed during these censuses are similar to the semiannual state count methods in which all waterbirds observed or heard during the survey are counted. The data collected during Refuge surveys provide an index of year-round Refuge occupancy by the waterbirds that is not attainable from the semiannual state counts.

### Data Analysis

To establish long-term population trends and seasonal use, we used data from the semiannual waterbird counts for 1986–2007 (dataset available from State of Hawai'i Division of Forestry and Wildlife upon request) [14] and from the monthly Refuge counts for approximately the same time period (1989–2013; dataset available from authors upon request). This time period was chosen because it represents the most complete set of data and corresponds to the time when active management was implemented on Refuges. An initial evaluation of the semiannual count dataset identified some discrepancies/errors during certain sampling periods. Errors included asynchronous data collection or missing data for multiple or key wetlands (see also Reed et al. [15] for an expanded discussion of this database and its limitations). We removed sampling periods with these errors from our analysis. Table 1 identifies the final set of data used to complete both the long-term trend and seasonal use analyses.

**Table 1:** Waterbird data included in analyses.

Location	Years with Usable Data for Population Trend Analyses^a^
*Statewide Summer Data*	1986,1987,1989–1993,1995,1998–2001,2003–2006
*Statewide Winter Data*	1987,1990,1993,1995,1999–2002,2004–2007
	**Years with Data for Seasonal Occupancy Analysis** b
*James Campbell NWR*	1989–2008 *(n* = 328)
	Monthly:1989–1992
	Bi-Monthly: 1992–2008
*Pearl Harbor NWR*	1989–2008 *(n* = 344)
	Monthly:1989–1992
	Bi-Monthly: 1992–2008
*Kealia Pond NWR*	1994–2007 (*n* = 305)
	Bi-Monthly: 1994–2007
*Hanalei NWR*	2010–2013 (n = 40)
	Monthly: 2010–2013
*Huleia NWR*	2010–2012 (n = 30)
	Monthly: 2010–2012
*Kakahai*'*a NWR*	None

a Data for the long-term population trend analyses includes data collected during semiannual waterbird counts across the state (Refuge and non-Refuge wetlands) for years spanning 1986–2007.

b Data for the seasonal occupancy analysis includes data collected only on Refuges for years spanning 1989–2013.

We analyzed the semiannual waterbird count data from 1986–2007 to identify the statistically significant long-term population trends across the state (all Refuge and non-Refuge wetlands), and on Refuge and non-Refuge wetlands independently. For the Hawaiian Coot, Gallinule, and Stilt we assessed long-term population trends and indices using Program TRIM Version 3.53 [16], which computes indices and trends for time series by means of log-linear Poisson regression and accounts for missing data points. Serial correlations were considered in TRIM with a Generalized Estimating Equations approach. We calculated the annual index and long-term population trend with standard errors for each species using the entire statewide database, data for Refuges only, and data excluding Refuges during the time period spanning 1986–2007. We estimated trends using the multiplicative trend, which reflects the average percent change per year [17]. The analysis started with a model incorporating change at each time point, and used a stepwise selection procedure to identify significant changes in slope based on Wald tests with a significance-level threshold value of p<0.05 [16]. All models were run with serial correlation considered, and a stepwise process was used to identify the best model.

We evaluated waterbird seasonal use patterns on Refuges by performing an analysis of variance and two multiple comparisons procedures on monthly Refuge count data spanning 1989–2013. We used Tukey's honest significant differences and pairwise t-tests with a holm adjustment in R Stats version 2.12.1 [18] to detect monthly differences in use of selected Refuges for each species. We identified significant differences as those that had a p-value of p<0.05.

Finally, to understand the relative importance of the actively managed Refuges for each of the endangered waterbird species, we averaged the totals of the two semiannual statewide counts by species and year to obtain an annual population estimate. We then averaged the corresponding data for Refuges and calculated what percent of the total species' population occurred on Refuges for each species by year.

## Results

We found that populations of the Hawaiian Coot, Stilt, and Gallinule were either stable or increasing across the state, and on both Refuges and non-Refuge wetlands when analyzed independently (Table 2). For Hawaiian Coots, the statewide population trend indicated an average population increase of 0.18% per year between 1986 and 2007.The population growth rate was −0.56% per year on Refuge wetlands and 0.83% for non-Refuge wetlands. The population trend, following criteria in TRIM, was identified as ‘stable’ (no significant increase or decline, and it is certain that trends are less than 5% per year) statewide and for both Refuge and non-Refuge wetlands. The statewide estimated average Hawaiian Coot population size was 1,777±310 for the subset of data used spanning 1986–2007. State population estimates for Hawaiian Coots ranged from>2,500 in 1998 to∼1,200 coots in 2002.

**Table 2:** Annual population trends for the Hawaiian Coot, Stilt, and Gallinule from count data spanning 1986–2007 in Hawai'i, USA.

Location^a^	Trend	SE	Interpretation^b^
*Refuge Gallinule*	1.0546	0.0202	Moderate Increase
*Refuge Coot*	0.9944	0.0046	Stable
*Refuge Stilt*	1.0311	0.0040	Moderate Increase
*Non-Refuge Gallinule*	1.0779	0.0049	Moderate Increase
*Non-Refuge Coot*	1.0083	0.0049	Stable
*Non-Refuge Stilt*	1.0125	0.0013	Moderate Increase
*State Gallinule*	1.0686	0.0097	Moderate Increase
*State Coot*	1.0018	0.0037	Stable
*State Stilt*	1.0198	0.0021	Moderate Increase

a Estimates by species for the entire state population (Refuge and non-Refuge lands combined); population on Refuges; and population on non-Refuge lands.

b As defined by program TRIM [16], [17].

For Hawaiian Stilts, the statewide population trend indicated an average population increase of 1.98% per year between 1986 and 2007. The population growth rate was 3.11% per year on Refuge wetlands and 1.25% on non-Refuge wetlands. The population trend, following criteria in TRIM, was identified as ‘moderate increase’ (any significant trend increase<20% per year) statewide and for both Refuge and non-Refuge wetlands. State population estimates peaked at∼2,200 stilts in our most recent estimate (2007).

For Hawaiian Gallinules, the statewide population trend indicated an average population increase of 6.86% per year between 1986 and 2007. The population growth rate was 5.46% per year on Refuge wetlands and 7.79% on non-Refuge wetlands. The population trend, following criteria in TRIM, was identified as ‘moderate increase’ (any significant trend increase<20% per year) statewide and for both Refuge and non-Refuge wetlands. State population counts exceeded∼400 Gallinules in 2005. Results showing stable to increasing long-term trends for all three species are similar to those found by other recent analyses of similar data [15], [19].

**Figure 2:**
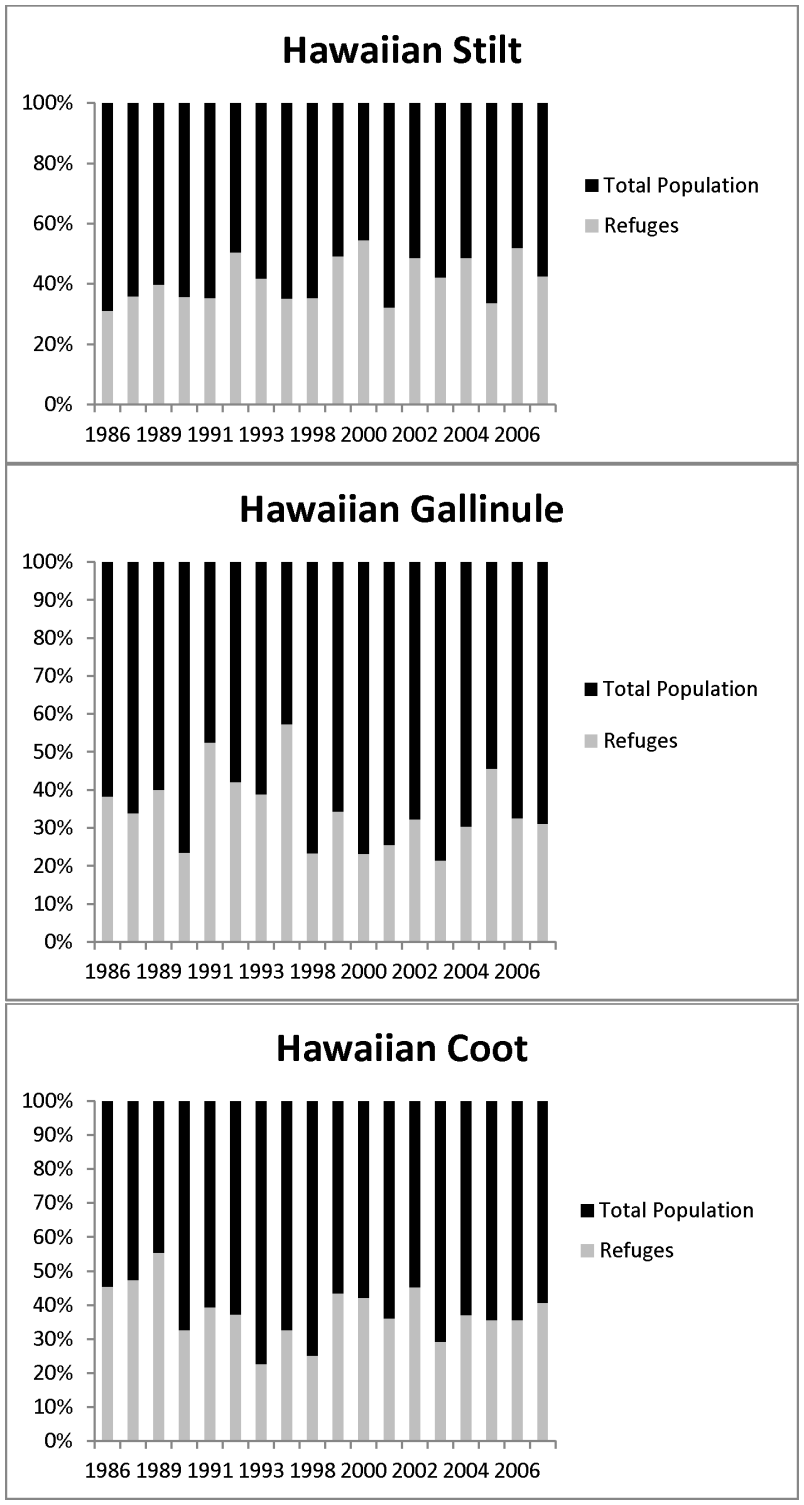
Percentage of endangered waterbird populations counted on six national wildlife refuges in Hawai'i, USA for the time period spanning 1986–2007. Refuges account for only 15% of available wetland habitat.

Although Refuges contain 15% of the total coastal plain wetland acreage in the state they support a disproportionate percentage of each waterbird species' population (see Figure 2). For Hawaiian Coots, the estimated average percentage of the statewide population found on Refuges for the subset of data used spanning 1986–2007 was 35.7%±4.7%. For Hawaiian Stilts, the estimated average percentage of the statewide population found on Refuges for the subset of data used spanning 1986–2007 was 41.5%±5.0%. For Hawaiian Gallinule, the estimated average percentage of the statewide population found on Refuges for the subset of data used spanning 1986–2007 was 33.7%±6.6%. When we assessed the trend for the relative contribution of the Refuges over time, we found that the pattern has remained stable.

**Figure 3:**
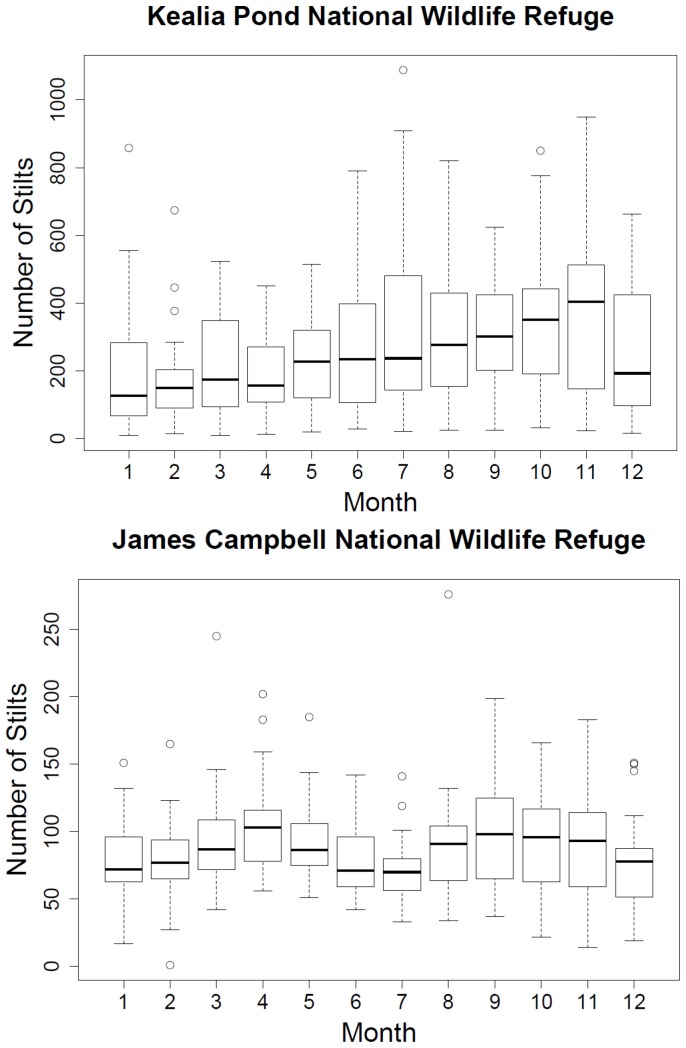
Seasonal use by Hawaiian Stilts on two national wildlife refuges in Hawai'i, USA for the time period spanning 1989–2008. The box represents the upper (75%) and lower (25%) quartiles, the horizontal line in the box shows the median, and vertical dashed lines (whiskers) indicate either the maximum and minimum values or 1.5 times the interquartile range (2 standard deviations). Data beyond the end of the whiskers are outliers and plotted as hollow circles.

**Figure 4:**
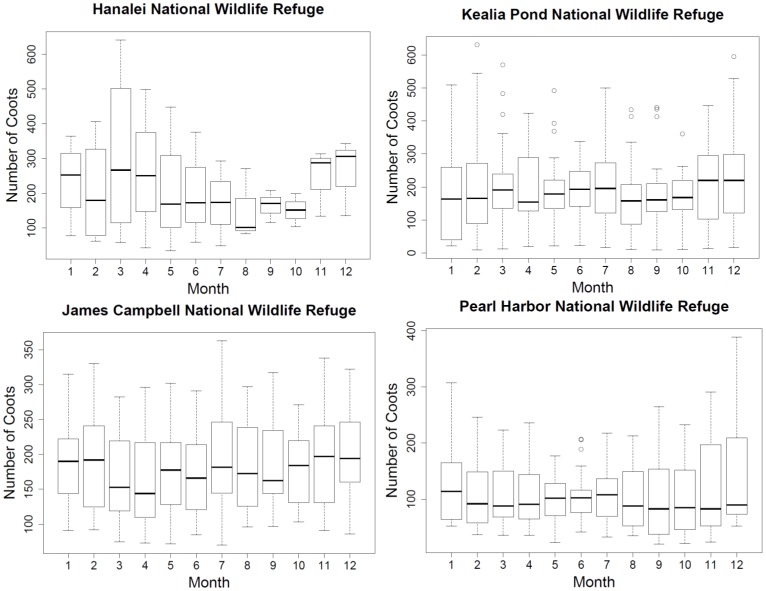
Seasonal use by Hawaiian Coots on four national wildlife refuges in Hawai'i, USA for the time period spanning 1989–2013. The box represents the upper (75%) and lower (25%) quartiles, the horizontal line in the box shows the median, and vertical dashed lines (whiskers) indicate either the maximum and minimum values or 1.5 times the interquartile range (2 standard deviations). Data beyond the end of the whiskers are outliers and plotted as hollow circles.

We found that Refuge wetlands are used year-round by all three species. Some patterns of seasonal use were detected that differed between Refuges. Significantly fewer Hawaiian Stilts were found to be using James Campbell NWR during July when compared to April (p<0.05; see Figure 3). At Kealia Pond NWR, Hawaiian Stilts occurred in significantly less numbers in February than in October and November (p<0.04, p<0.05; see Figure 3). Hawaiian Coots (see Figure 4) and Gallinules did not exhibit any significant pattern of seasonal use.

## Discussion

The status of the endangered Hawaiian Coot, Gallinule, and Stilt appears to be improving. All three species have increased in relative abundance over the last several decades and have rebounded from historic lows. We found that populations statewide and on Refuges have remained stable or have increased. Based on our metric of relative importance, we would conclude that the current active management strategy is successful as populations of all three species are increasing or stable, and a disproportionate number of birds are found on Refuges. That said, the relative importance of Refuges stayed the same over the time period analyzed and populations of two of the species are actually growing at a faster rate off Refuge lands. This is potentially the result of many factors, but we believe that this also corroborates the finding that the current active management strategy is successful because over the last two decades there has been an incremental increase in active management of wetlands for waterbirds both on Refuges and by other federal, state, local, private, and non-governmental conservation partners with wetland management responsibilities.

Populations off Refuge might also have increased at a faster rate as active management capacity on some Refuge wetlands remained fairly constant during the time period examined. Without active management of large additional areas of wetland on Refuges, the number of quality territories likely remained similar from year to year and young birds may have dispersed to non-Refuge wetlands. Limited band resights have documented Refuges as important source populations which contribute to populations in wetlands across the main Hawaiian Islands [20]. However, while the number of birds documented occupying non-Refuge wetlands may be increasing at a faster rate for the Hawaiian Coot and Gallinule, this does not mean birds in these wetlands are contributing to the long-term species viability in an equal manner. Although reproductive success is not routinely monitored at many of the unmanaged or minimally managed wetlands, occasional assessments of reproductive success on these wetlands have documented little to no reproductive success even when population counts are in the hundreds [21], [22]. This finding highlights the importance of the active management strategy implemented on Refuge and other wetlands for the long-term viability of these endangered waterbird species.

### Current Management Strategy

The current active management strategy appears to be successful at maintaining and even increasing populations for several of these endangered species. To meet the recovery goals for these species an active management strategy would need to be implemented on all key wetlands in the state identified in the Recovery Plan of Hawaiian Waterbirds [2]. This would result in expansion of the active management strategy to an additional 1,000–2,000 ha of wetlands [2]. Implementing such an action would require a sizeable commitment of both time and money, estimated in the Recovery Plan of Hawaiian Waterbirds [2] to be at least 2–3 million USD annually. Since current active management efforts are often time consuming and expensive, use of adaptive management frameworks should be expanded so that managers can identify which active management actions provide the greatest benefit to endangered waterbirds.

It is our belief that without perpetual active management of wetlands in Hawai'i, the endangered waterbird species likely would experience population declines. As an example of what can happen when a wetland is not actively managed, Kawainui Marsh on the island of Oahu is one of the largest remaining natural wetlands in Hawai'i, totaling approximately 400 ha. It was named as a Wetland of International Importance under the RAMSAR convention in 2005 [23]. Historically it was used by hundreds of endangered waterbirds and migratory waterfowl [24], [25], [26]. However, growth of invasive plants and sedimentation resulting from altered hydrology had resulted in almost a complete loss of wetland functionality for waterbirds. As a result, one of the largest natural wetlands in the state provided habitat for only a handful of birds. Fortunately, in 2012 the State of Hawai'i and the U.S. Army Corp of Engineers began projects to restore a portion of this wetland to a functional state. Post restoration, this portion of the wetland will be actively managed for endangered waterbirds. When such restoration actions occur, we have observed on Refuges that populations of endangered waterbirds in the restored areas have increased dramatically (unpublished data). We believe that wetland restoration efforts both on and off Refuges are a major contributor to the increasing populations of endangered waterbirds.

In addition to the current threats of weedy invaders, disease, predation, and hybridization faced by the waterbirds, most wetlands used by these endangered waterbirds are also threatened with the impacts of sea level rise. Estimates of sea level rise range from 20 to 190 cm in Hawai'i by the end of the 21^st^ century [27], [28]. Sea level rise is projected to result in the loss of some wetland habitat and is expected to negatively impact the habitat quality of other wetlands by increasing salinity levels [29], [30], [31]. Increased salinity would have a negative impact on species that prefer freshwater such as the Hawaiian Duck and Gallinule [2]. Strategies to mitigate for the loss of wetlands and wetland functionality due to sea level rise include the restoration of wetlands located above the new projected sea level or the creation of additional wetlands in similar locations. To effectively manage these species into the future, the current active management paradigm must be expanded to consider the impacts of sea level rise.

#### Caveats

We acknowledge that the data used for this analysis may have errors for a variety of reasons. First, bird detectability is somewhat contingent on vegetative cover that can differ among wetlands. As surveys are reliant mainly on visual counts to estimate population size, locations with dense vegetative cover can obscure the true number of birds leading to underestimation. Second, the effort expended (amount of time and number of census stations) also varies between wetlands and can lead to differences in estimation. Third, observer experience or familiarity with the wetland being counted may have caused some differences. For example, Refuge biologists often have a more intimate familiarity with the wetlands they manage compared to surveyors that visit wetlands only for the semiannual counts. This may allow Refuge biologists to have a better idea of how/where to find the birds, resulting in more comprehensive counts. Lastly, the general survey methodology needs to be improved through greater standardization and consistency among islands in the identification of Hawaiian Ducks, feral Mallards, and their hybrids and more consistent coverage of wetlands each year to increase comparability over time. Implementation of more accurate methods of surveying for Hawaiian Gallinules (call counts), and inclusion of montane stream habitats used by Hawaiian Ducks would provide more accurate population estimates for theses species [2], [8], [11]. Finally, the feasibility of alternative methods such as point counts should be explored.

## Conclusion

To maintain the recent increases in endangered Hawaiian waterbird populations, we will need to continue to improve both the quality and quantity of available wetland habitat. It is likely that the fate of these species will be perpetually tied to the amount of habitat that is actively managed for them. In addition to all currently required active management activities, it will also likely be necessary to mitigate for the loss of wetland habitat as a result of sea level rise. While the costs of active wetland management are not trivial, the endangered waterbirds of Hawai'i currently represent some of the only endangered Hawaiian species for which we have been able to reverse the population trajectory on a statewide scale. As a result, they represent one of the best returns on investments and conservation successes demonstrable in Hawai'i.
